# The Impact of Simvastatin on Pulmonary Effectors of *Pseudomonas aeruginosa* Infection

**DOI:** 10.1371/journal.pone.0102200

**Published:** 2014-07-10

**Authors:** Emma Hennessy, Julie O'Callaghan, Marlies J. Mooij, Claire Legendre, Olga Camacho-Vanegas, Sandra C. Camacho, Claire Adams, John A. Martignetti, Fergal O'Gara

**Affiliations:** 1 BIOMERIT Research Centre, School of Microbiology, National University of Ireland, Cork, Ireland; 2 Department of Genetics and Genomic Sciences, Icahn School of Medicine at Mount Sinai, New York, New York, United States of America; 3 School of Biomedical Sciences, Curtin University, Perth, WA, Australia; Harvard Medical School, United States of America

## Abstract

The statin family of cholesterol-lowering drugs is known to have pleiotropic properties which include anti-inflammatory and immunomodulatory effects. Statins exert their pleiotropic effects by altering expression of human immune regulators including pro-inflammatory cytokines. Previously we found that statins modulate virulence phenotypes of the human pathogen *Pseudomonas aeruginosa*, and sought to investigate if simvastatin could alter the host response to this organism in lung epithelial cells. Simvastatin increased the expression of the *P. aeruginosa* target genes KLF2, KLF6, IL-8 and CCL20. Furthermore, both simvastatin and *P. aeruginosa* induced alternative splicing of KLF6. The novel effect of simvastatin on wtKLF6 expression was found to be responsible for induction of the KLF6 regulated genes CCL20 and iNOS. Simvastatin also increased the adhesion of *P. aeruginosa* to host cells, without altering invasion or cytotoxicity. This study demonstrated that simvastatin had several novel effects on the pulmonary cellular immune response.

## Introduction

Statins are a class of drug of which the major medical use is the inhibition of cholesterol biosynthesis. However, statins also have cholesterol-independent pleiotropic effects, including the modulation of genes involved in the immune and inflammatory responses, which in turn lead to overall protective effects against infections and cancer [1]–[3]. In particular, statins are known to have protective effects against bacterial infections. It has previously been demonstrated that statins improved survival in patients who had sepsis and pneumonia [1], [4]–[6].

One key effect of statin treatment is the inhibition of inflammatory pathways. For instance, statins reduced activation of the pro-inflammatory cytokine Interleukin-8 (IL-8) by bacterial and fungal surface antigens in *ex vivo* blood cells [7]. Production of another infection-induced chemokine, Chemokine (C-C motif) ligand 20 (CCL20; also known as macrophage-inhibitory protein 3α (MIP-3α)), was also reduced by statin treatment of keratinocytes [8]. As well as inhibiting pro-inflammatory components, statins also increase the expression of anti-inflammatory components, such as endothelial nitric oxide synthase (eNOS) [9] and thrombomodulin [10]. The anti-inflammatory and immunomodulatory effects of statins are mediated through the alteration of expression of transcription factors such as NFκB [11]. Interestingly, despite the fact that the majority of studies involving statins and inflammation describe an anti-inflammatory effect, several studies have reported pro-inflammatory effects of statins [12]–[15].

One group of transcriptional regulators that is now emerging as both a key group of immune and inflammatory regulators and a target of statin treatment is the Kruppel-like Factors (KLFs). These are a family of transcriptional regulators characterised by the presence of 3 conserved zinc fingers [16]. We have previously found 2 KLF family members, KLF2 and KLF6, to be increased in an *in vitro Pseudomonas aeruginosa* infection model [17]. *P. aeruginosa* is an important human pathogen that causes serious infections in immunocompromised individuals and is the main pathogen associated with chronic refractory pulmonary infections in cystic fibrosis (CF) patients. The pathogenesis of *P. aeruginosa* can be attributed to a plethora of virulence associated phenotypes including the production and secretion of toxins via the Type 3 secretion system (T3SS). Previously we have shown that the exotoxins ExoS and ExoY secreted by the T3SS are required for *P. aeruginosa* induction of KLF2 and KLF6 in CF airway epithelial cells [17]. KLF2 is mainly characterised as an anti-inflammatory regulator which can control the activation of monocytes and macrophages [18], [19], and has been found to be induced by toxin activity of *Staphylococcus aureus* [20], *Yersinia enterocolitica* [21] and *Clostridium botulinum* [22]. Furthermore, statins can also induce the expression of KLF2 [22], [23].

The role of KLF6 in infections is currently less clear than that of KLF2. KLF6 is most widely-recognised as a tumour suppressor protein which inhibits proliferation [24], and induces apoptosis [25]. However, KLF6 is known to be alternatively spliced into a full-length transcript (wtKLF6) and 3 truncated variants (SV1, SV2, SV3) [26]. In contrast to the tumour suppressive action of wtKLF6, the splice variants of KLF6, particularly SV1, have been found to have oncogenic properties and have been implicated in several types of cancer including lung, liver, ovarian and prostate [26]–[29]. Given that they do not possess the 3 zinc fingers of wtKLF6 it is believed the splice variants cannot exert their biological effects through direct binding of DNA but instead must interact with other proteins [30]. For example, SV1 has been shown to bind directly to wtKLF6 accelerating its degradation and thus antagonising its tumour suppressor function [31].

While to date, it has not been characterised, it is likely that KLF6 does play a role in bacterial infections, as it is upregulated by *S. aureus* [20] and *P. aeruginosa* [17], and can promote the apoptosis of cells infected with respiratory syncytial virus [32] and influenza A [33]. Furthermore, some regulatory targets of KLF6 are known to have roles in the immune response to infections. KLF6 directly activates promoter expression of ASAH1 (acid ceramidase) [34] and iNOS (inducible nitric oxide synthase; NOS2) [35], two proteins that regulate the production of signalling molecules with roles in the inflammatory response. ASAH1 regulates the production of ceramide, which is upregulated as part of the host response to *P. aeruginosa* [36], leading to increased inflammation [37]. iNOS is responsible for the production of nitric oxide (NO), a signalling molecule which is responsible for increased inflammation, lung damage and bacterial killing during infections [38], [39]. In addition, KLF6 also induces the expression of the pro-inflammatory chemokine CCL20 (MIP-3α). It has been proposed that this effect is indirect, mediated through the KLF6-dependent inhibition of another transcription factor called Peroxisome Proliferator-Activated Receptor γ (PPARγ) in kidney cells [40].

In this study, we sought to investigate the effect of simvastatin on the expression of *P. aeruginosa*-responsive immune modulators, including KLF6 splice variants, in a lung epithelial cell model. In addition to KLF2 and KLF6, *P. aeruginosa* has been shown to alter the expression of several modulators of the host immune response including IL-8 [41], CCL20 [42] and TLR5 (Toll-like Receptor 5) [43], which activates NFκB-dependent inflammation in response to bacterial cell surface component flagellin [44], [45]. We demonstrate that simvastatin induces pro-inflammatory modulators of the immune response and that both *P. aeruginosa* and simvastatin induce KLF6 splice variants in A549 epithelial cells, with the wtKLF6 being the dominant variant. Thus, we observed that simvastatin could alter the genetic and physiological immune response in lung cells and KLF6 was identified as a novel target of statins and an important transcriptional regulator in the lung.

## Materials and Methods

### Mammalian cells and bacterial strains

All cell lines and bacterial strains used in this study are detailed in Table 1. A549 cells (ATCC) were grown in minimal Eagle's medium (MEM) (Sigma-Aldrich, U.K.) supplemented with 10% FBS, 50 units/ml pen-strep and 2 mM L-glutamine. Infection assays were carried out using co-culture medium (MEM minus pen-strep), and cells that were used for determination of cytotoxicity were cultured in serum-free MEM. All media components were obtained from Gibco unless stated otherwise. Cells were incubated at 37 °C in a humidified 5% CO_2_ atmosphere. *P. aeruginosa* was cultured in Luria-Bertani (LB) broth (10 g/L tryptone, 5 g/L yeast extract, 5 g/L NaCl) and were subcultured for infection assays in co-culture medium.

**Table 1:** Cells and bacteria used in this study.

	Type	Source
**Mammalian cell line**		
A549	Wild type squamous lung epithelia	ATCC
A549 pSUPER-wtKLF6	si-RNA-mediated knockdown of wtKLF6	This study
A549 pSUPER-Retro-luc	Vector Control	This study
**Bacteria**		
*Pseudomonas aeruginosa* PAO1	Wild type	[65]

### Generation of stable cell lines for knockdown of wtKLF6

Stable cell lines were generated by retroviral infection of A549 cells with a pSUPER retroviral vector containing either siRNA specifically designed against wtKLF6 (pSUPER si-wtKLF6) or luciferase as control (pSUPER-luc). These retroviral vectors have been previously described [46]. For standard infection, approximately 1×10^6^ viral particles were incubated with 4.8×10^5^ A549 cells in a final volume of 6 ml in the presence of 10 µg/ml polybrene (Sigma) for 3 successive 12 hour periods. Infected cells were selected using 0.5 µg/ml puromycin (Sigma) and used for subsequent analysis.

### Statin treatment and infection of cells

Simvastatin (Sigma-Aldrich) was resuspended in DMSO at a concentration of 20 mg/ml, filter sterilised and stored at 4 °C. Cells were trypsinised, counted and seeded into plastic vessels. Cells were then incubated until they achieved 80% confluency following which they were treated with either 10 µM simvastatin or an equivalent volume of DMSO for 24 hours. Bacterial strains were cultured shaking at 37 °C at 16–18 hours in LB broth, following which they were sub-cultured in infection medium shaking at 37 °C for 3 hours. Bacterial strains were centrifuged (2900× *g*) and washed twice with phosphate-buffered saline (PBS), following which bacterial densities were adjusted so as to infect cells at a multiplicity of infection (MOI) of approximately 50∶1.

### RNA isolation and quantitative real-time PCR (qRT-PCR)

RNA was isolated using an RNeasy mini kit (QIAGEN, Germany) according to manufacturer's specifications, and was quantified using a ND-1000 Spectrophotometer (NanoDrop Technologies, USA). Genomic DNA was removed using Turbo DNA-*free* (Ambion), and samples were confirmed to be free of DNA by PCR. RNA was converted to cDNA using oligo-d(T) and AMV reverse transcriptase (both Promega) according to manufacturer's instructions. qRT-PCR was carried out using the Universal ProbeLibrary (UPL) system (Roche) according to manufacturer's specifications, and samples were normalised to hypoxanthine phosphoribosyltransferase (HPRT). A full list of the primers and UPL probes used in this study is detailed in Table 2.

**Table 2:** Primers used for qRT-PCR analysis of host gene expression.

Gene	Product Length (bp)	Sequence	Probe No. *
HPRT	102	Forward: tgaccttgatttattttgcatacc	73
		Reverse: cgagcaagacgttcagtcct	
KLF2	67	Forward: cctcccaaactgtgactggt	115
		Reverse: ctctgtagccacgctgtgc	
wtKLF6	70	Forward: aaagctcccacttgaaagca	2
		Reverse: ccttcccatgagcatctgtaa	
SV1	109	Forward: ggcacttccgaaagcaca	24
		Reverse: cctcagaggtgcctcttcat	
SV2	96	Forward: gggaaccttctcaactgtgg	24
		Reverse: aaggcttttctcctggcttc	
SV3	96	Forward: acgcacacaggtgtttttcc	32
		Reverse: ccttttagcctacaggatccac	
IL-8	77	Forward: gccaggatccacaagtcct	98
		Reverse: tggtggctaatactttttccact	
CCL20	77	Forward: tggcttttctggaatggaat	117
		Reverse: tgtgcaagtgaaacctccaa	
ASAH1	86	Forward: ttgacatttggggatctggt	10
		Reverse: attcaacacccacgctgaa	
iNOS	68	Forward: ttccttacgaggcgaagaag	3
		Reverse: tcagagcgctgacatctcc	
PPARγ	78	Forward: accagctgaatccagagtcc	81
		Reverse: gcgggaaggactttatgtatga	

* Roche Universal ProbeLibrary (UPL) Probe Number.

The annealing temperature of all primer sets is 60°C.

### Determination of cytotoxicity

Cytotoxicity of statin treatment and *P. aeruginosa* infection was measured using a LDH Cytotoxicity Detection Kit (Roche) according to manufacturer's instructions. Infection and statin-treatment of A549 cells were set up as previously described, except that in this instance serum-free medium was used for infection as FBS caused high background levels of LDH activity. The OD_492nm_ of samples was measured using a SpectraMax Plus 384 96 well plate reader. Five independent biological replicates of each experiment were carried out.

### Measurement of bacterial adhesion and invasion

Adhesion and invasion of bacteria were quantified as described by Burns *et al.* [47]. Briefly, A549 cells were treated with simvastatin and infected with *P. aeruginosa* PAO1 as previously described. For determination of invasion, after 1 hour of infection ceftazimide (1 mg/ml) and gentamicin (2 mg/ml) (both Sigma-Aldrich) were added for a further 2 hours, following which cells were washed twice with PBS and lysed using 0.1% Triton X-100. Intracellular bacteria were enumerated by serial dilutions and plate counts. To measure bacterial adherence, extracellular bacteria were removed from epithelial cells, which were lysed to provide a total and intracellular count following 3 hours of infection. The adherent count was determined by subtracting the number of intracellular and extracellular bacteria from the total bacterial count. Five independent biological replicates were performed for each experiment.

### Statistical analysis

Three independent biological replicates were performed for each experiment unless otherwise stated. Statistical significance was measured using a paired two-tailed Student's T-test. Differences were considered to be statistically significant if the p-value was ≤0.05.

## Results

### Simvastatin alters the gene expression of *P. aeruginosa*-responsive host immune modulators

**Figure 1:**
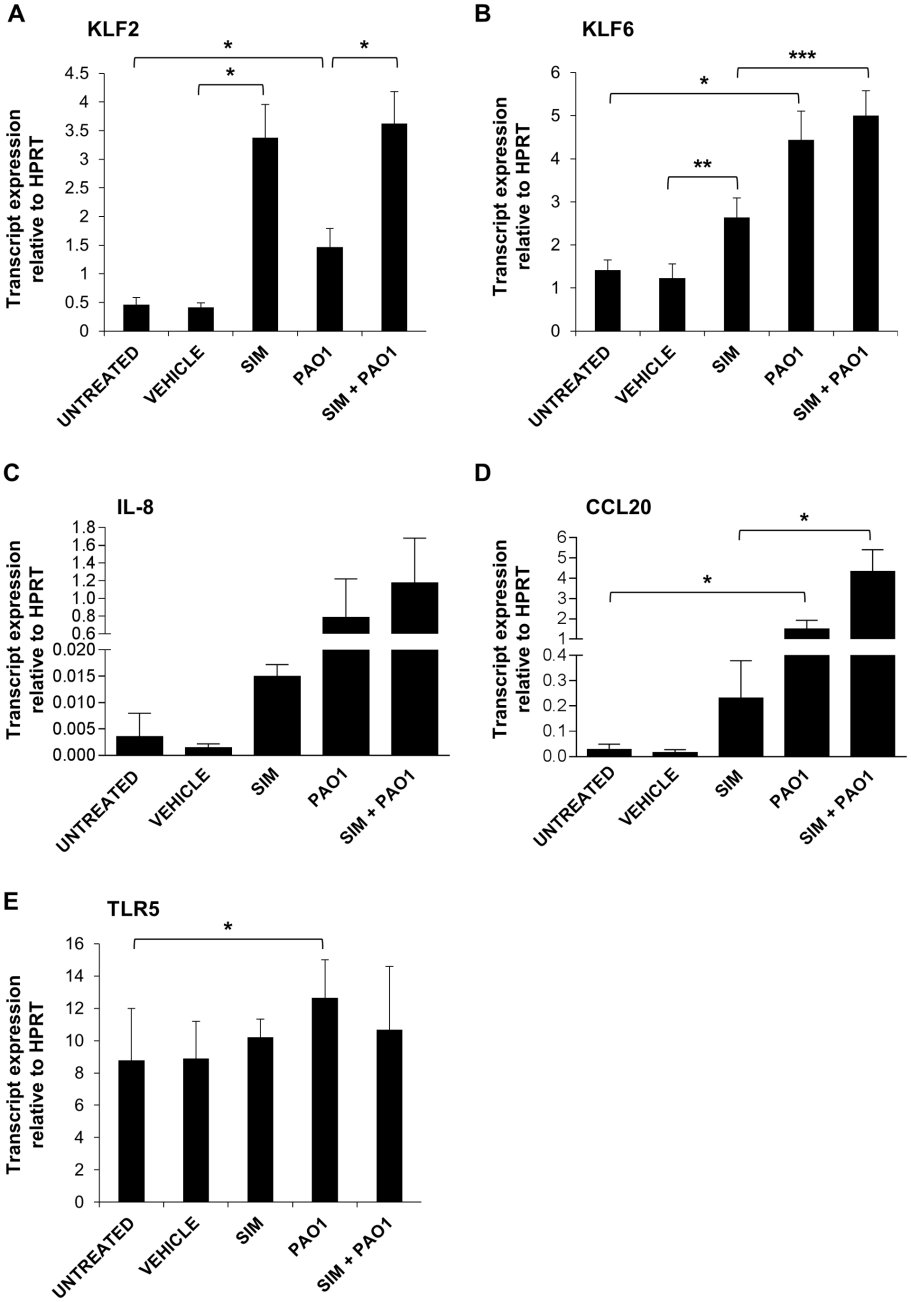
Statin treatment and *P. aeruginosa* infection modulate the host immune response. A549 cells were treated with 10 µM simvastatin or an equivalent amount of DMSO (VEHICLE) for 24 hours. Untreated and statin-treated cells were subsequently infected with *P. aeruginosa* PAO1 for 3 hours at MOI 50∶1. The expression of (**A**) KLF2 (**B**) KLF6 (**C**) IL-8 (**D**) CCL20 and (**E**) TLR5 was analysed in uninfected cells, and in the presence of vehicle, simvastatin (SIM), *P. aeruginosa* (PAO1) and simvastatin and PAO1 combined (SIM+PAO1). *P, ≤0.05, **P, ≤0.01, ***P, ≤0.001.

In order to investigate the effect of statins on the expression of infection-responsive, immunomodulatory genes, 5 genes previously shown to be induced during *P. aeruginosa* infection were selected for analysis; KLF2, KLF6, IL-8, TLR5 and CCL20 [13], [37]–[39]. The expression of these candidate genes was analysed in A549 squamous epithelial lung cells which were treated with 10 µM simvastatin or an equivalent volume of DMSO vehicle control for 24 hours, following which they were infected with *P. aeruginosa* PAO1 at an MOI of 50∶1 for 3 hours. Gene expression was analysed using qRT-PCR. Simvastatin has been found to induce KLF2 in endothelial cells & human peripheral blood monocytes [22] and our model complied with these findings. KLF2 was significantly increased in statin-treated A549 cells compared to DMSO-treated cells (*P* = 0.012) (Figure 1A). Interestingly, *P. aeruginosa* also significantly induced KLF2 expression (*P* = 0.033) but to a much lesser degree than simvastatin alone and there was no additive effect in statin-treated infected cells (*P* = 0.008). Previous studies have shown that statins did not have an effect on KLF6 expression [22], however, here we demonstrate a significant induction of wtKLF6 by simvastatin treatment in A549 lung epithelial cells (*P* = 0.005) (Figure 1B). *P. aeruginosa* also significantly induced KLF6 (*P* = 0.015), but to a greater extent than simvastatin, and this effect was sustained in simvastatin-treated infected cells (*P* = 0.0019). Thus, there was also no additive effect of simvastatin and *P. aeruginosa* on wtKLF6 expression.

As expected, *P. aeruginosa* infection substantially induced IL-8 expression; transcript levels in infected cells were over 100 times higher than control cells (Figure 1C). Interestingly, in contrast to published reports [7] we observed that simvastatin increased the expression of IL-8 by 14.75-fold compared to vehicle treated epithelial cells. However, when cells were pre-treated with simvastatin and subsequently infected with *P. aeruginosa*, the level of IL-8 expression was not significantly different to untreated infected cells. CCL20 expression was also significantly increased by *P. aeruginosa* compared to uninfected cells (*P* = 0.03) and it was increased to a lesser extent (*P* = 0.02) by simvastatin alone compared to vehicle-treated cells (Figure 1D). Interestingly however, in simvastatin-treated infected cells, a significant increase in CCL20 expression was observed (*P* = 0.02). This effect was greater than CCL20 induction by simvastatin (*P* = 0.02) or *P. aeruginosa* (*P* = 0.054) individually, suggesting that CCL20 expression was synergistically induced by a combination of these factors. In contrast to IL-8 and CCL20, TLR5 expression was not significantly altered by simvastatin (Figure 1E). The expression of this gene was significantly increased by *P. aeruginosa* (*P* = 0.015) but interestingly, under combined statin treatment and infection TLR5 expression levels were comparable to statin-treated uninfected cells, suggesting that simvastatin may reduce *P. aeruginosa*-mediated induction of TLR5.

### Novel induction of KLF6 alternative splicing by *P. aeruginosa* and simvastatin

**Figure 2:**
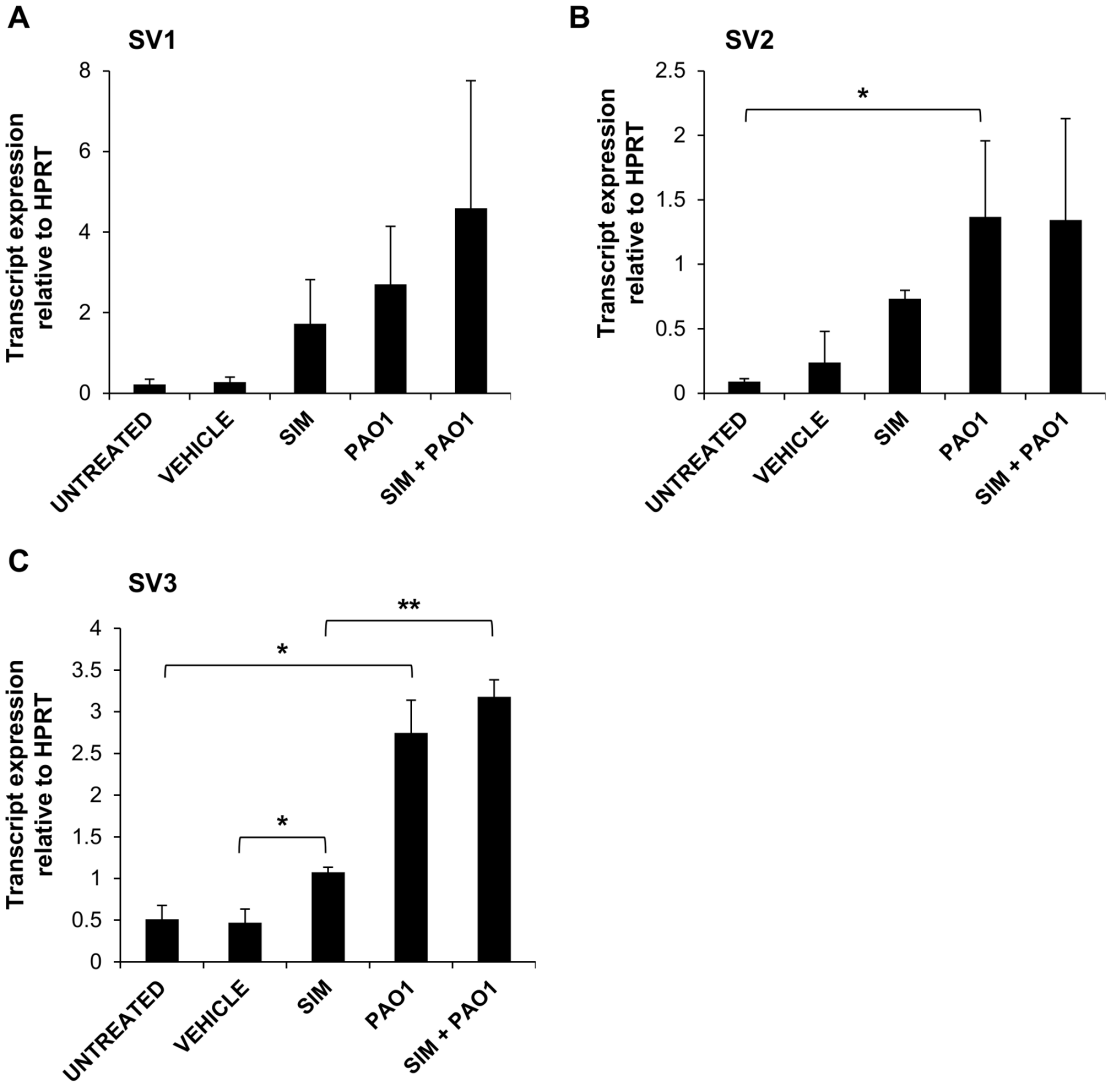
Induction of KLF6 splice variants by simvastatin. A549 cells were treated with 10 µM simvastatin for 24 hours, following which they were infected with *P. aeruginosa* PAO1 for 3 hours at MOI 50∶1. The expression of (**A**) SV1, (**B**) SV2 and (**C**) SV3 was examined using qRT-PCR. *P, ≤0.05, **P, ≤0.01.

While our results confirm *P. aeruginosa* induction of wtKLF6 expression [17], the expression of KLF6 splice variants in the context of bacterial infections has never been characterised. In addition, the impact of statins on KLF6 alternative splicing has yet to be investigated. Therefore, we sought to examine whether statins and *P. aeruginosa* could influence the alternative splicing of KLF6 in lung epithelial cells, using qRT-PCR (Figure 2). Simvastatin increased the expression of SV1 (Figure 2A), SV2 (Figure 2B) and SV3 (Figure 2C), with SV3 levels reaching significance (*P* = 0.01). *P. aeruginosa* induced the expression of all 3 KLF6 splice variants but to varying degrees. Of the 3 variants, SV3 was increased to the greatest extent (*P* = 0.005), and SV2 was also significantly induced (*P* = 0.013). SV1 displayed the lowest level of induction and this did not reach significance. As with wtKLF6, *P. aeruginosa* had a greater effect on the expression of all 3 splice variants than simvastatin. In the presence of simvastatin and *P. aeruginosa* combined, there was no significant alteration in SV1, SV2 and SV3 levels compared to those in untreated infected cells suggesting that statins and *P. aeruginosa* do not have a synergistic effect on KLF6 alternative splicing. Interestingly, the transcript levels of wtKLF6 (Figure 1B) were higher than those of SV1, SV2 and SV3 under all conditions, suggesting that wtKLF6 was the dominantly expressed variant in A549 cells and in response to simvastatin and *P. aeruginosa* infection.

### Elucidating the downstream effect of statin-mediated KLF6 induction

**Figure 3:**
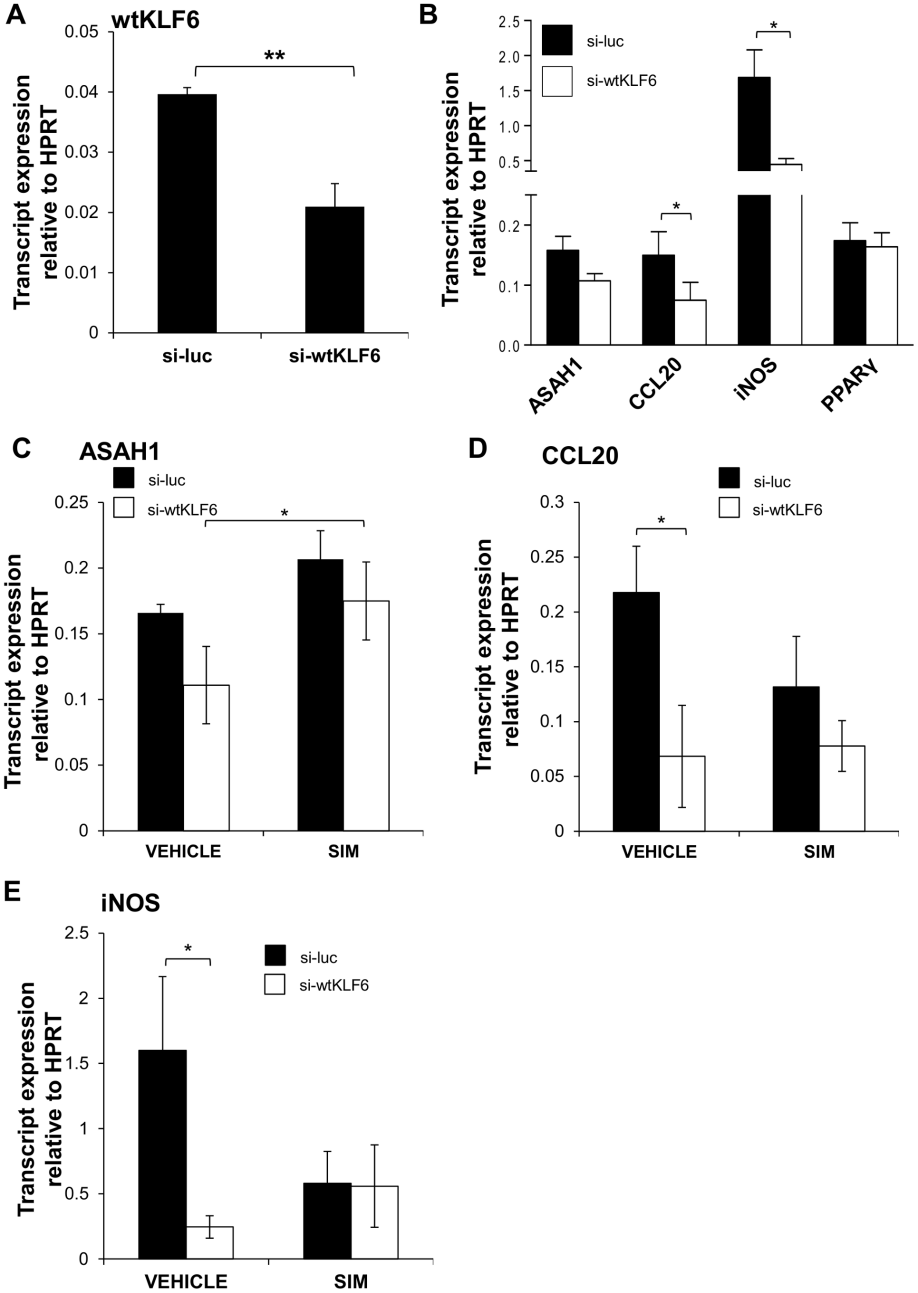
wtKLF6 knockdown and statin effects in lung epithelial cells. (**A**) wtKLF6 mRNA expression was reduced by 47.2% compared to vector control (si-luc) cells (*P* = 0.008). (**B**) Expression of the wtKLF6 target genes ASAH1, CCL20, iNOS and PPARγ was examined by qRT-PCR in si-wtKLF6 cells compared to vector control. ASAH1, CCL20 and iNOS were inhibited in si-cells compared to vector control cells. PPARγ was unaffected in si-wtKLF6. (**C**) Simvastatin induced ASAH1 expression but independent of wtKLF6. Simvastatin induced CCL20 (**D**) and reduced iNOS (**E**) expression and these appeared to be mediated via wtKLF6. *P, ≤0.05, ** *P*, ≤0.01.

In order to further examine the influence and impact of the novel induction of KLF6 by simvastatin, stable wtKLF6 knockdown cell lines (designated si-wtKLF6) were generated by infecting A549 cells with pSUPER si-wtKLF6, or a control cell line using the pSUPER si-luc vector (designated si-luc). To verify that the si-wtKLF6 cells were displaying reduced expression of wtKLF6, they were harvested for RNA isolation and the expression of wtKLF6 was analysed using qRT-PCR. It was demonstrated that in the si-wtKLF6 cells, wtKLF6 expression was significantly reduced by 47.2% compared to the cells containing the vector control (*P* = 0.008) (Figure 3A).

To investigate the consequences of wtKLF6 knockdown in lung epithelial cells, expression analysis was carried out on four KLF6 regulatory target genes; ASAH1, CCL20, iNOS and PPARγ. In si-wtKLF6 cells, expression of 3 of the target genes was attenuated (Figure 3B). ASAH1, CCL20 and iNOS expression was decreased by an average of 30.3% (non-significant), 51.5% (*P* = 0.012) and 72.9% (*P* = 0.047) respectively in si-wtKLF6 cells compared to vector control cells. However, the expression of PPARγ was comparable between si-wtKLF6 and vector control cells, suggesting that the expression of PPARγ in our model was not dependent upon wtKLF6. This also suggests that KLF6 regulation of CCL20 was not dependent on PPARγ, although it may be possible that the level of wtKLF6 reduction obtained in si-cells may not be enough to inhibit PPARγ expression.

Thus, wtKLF6 plays a key, multifaceted regulatory role in lung epithelia and we sought to examine the effect of simvastatin on wtKLF6 and its 3 regulatory targets (ASAH1, CCL20 and iNOS) in si-wtKLF6 lung cells. This analysis was carried out using qRT-PCR of transcripts isolated from si-wtKLF6 and si-luc cells which had been treated with 10 µM simvastatin for 24 hours. This analysis served the joint objectives of elucidating novel statin targets in the lung epithelial cells, and investigating the downstream effects of statin-mediated KLF6 induction.

Results demonstrated that simvastatin increased the expression of ASAH1 in si-luc control and si-wtKLF6 cells (*P* = 0.04) (Figure 3C). However, as its expression in statin-treated si-wtKLF6 cells was equivalent to that of statin treated si-luc control cells, the induction of ASAH1 by simvastatin did not appear to be mediated through wtKLF6. CCL20 expression analysis showed that the wtKLF6-dependent decrease in CCL20 that was observed in untreated si-wtKLF6 cells (Figure 3B) was maintained in vehicle-treated si-wtKLF6 cells (*P* = 0.04) (Figure 3D). However, in the presence of simvastatin CCL20 expression was decreased in vector control cells, implying that the vector may have had an effect on CCL20 expression under these conditions. Nevertheless, in si-wtKLF6 cells, the expression of CCL20 was equivalent between simvastatin-treated and DMSO-treated cells, suggesting that the novel simvastatin-mediated induction of CCL20 observed in A549 cells (Figure 1D) may be wtKLF6-dependent.

The third KLF6 target gene examined was iNOS. Interestingly, similarly to CCL20, simvastatin treatment reduced iNOS expression in si-luc control cells (Figure 3E). However, in si-wtKLF6 cells, this reduction was not observed, and expression was at an equivalent level to that of vector control cells. This latter observation suggests that simvastatin may inhibit iNOS expression in a KLF6-dependent mechanism.

### Cellular cytotoxicity and *P. aeruginosa* adhesion and invasion in response to simvastatin

Having established that simvastatin treatment can alter the expression of host immune-responsive genes, and having previously demonstrated the alteration of *in vitro P. aeruginosa* motility and early biofilm formation by statins [48], we investigated the effect of statins on *P. aeruginosa*-related cytotoxicity, adhesion and invasion of A549 epithelial cells. The cytotoxic effect of *P. aeruginosa* is exerted via its T3SS toxins, in particular ExoS [49]. ExoS in turn is required for *P. aeruginosa* induction of KLF6 [17] suggesting that KLF6 induction may contribute to *P. aeruginosa*-initiated cell death. Furthermore, the induction of IL-8 and CCL20 has previously been linked to bacterial adhesion and invasion [50]–[52] and therefore statins may have a regulatory impact on these phenotypes. To examine the effect of statins on these physiological outcomes, A549 cells were treated with 10 µM simvastatin for 24 hours and subsequently infected with *P. aeruginosa* PAO1.

**Figure 4:**
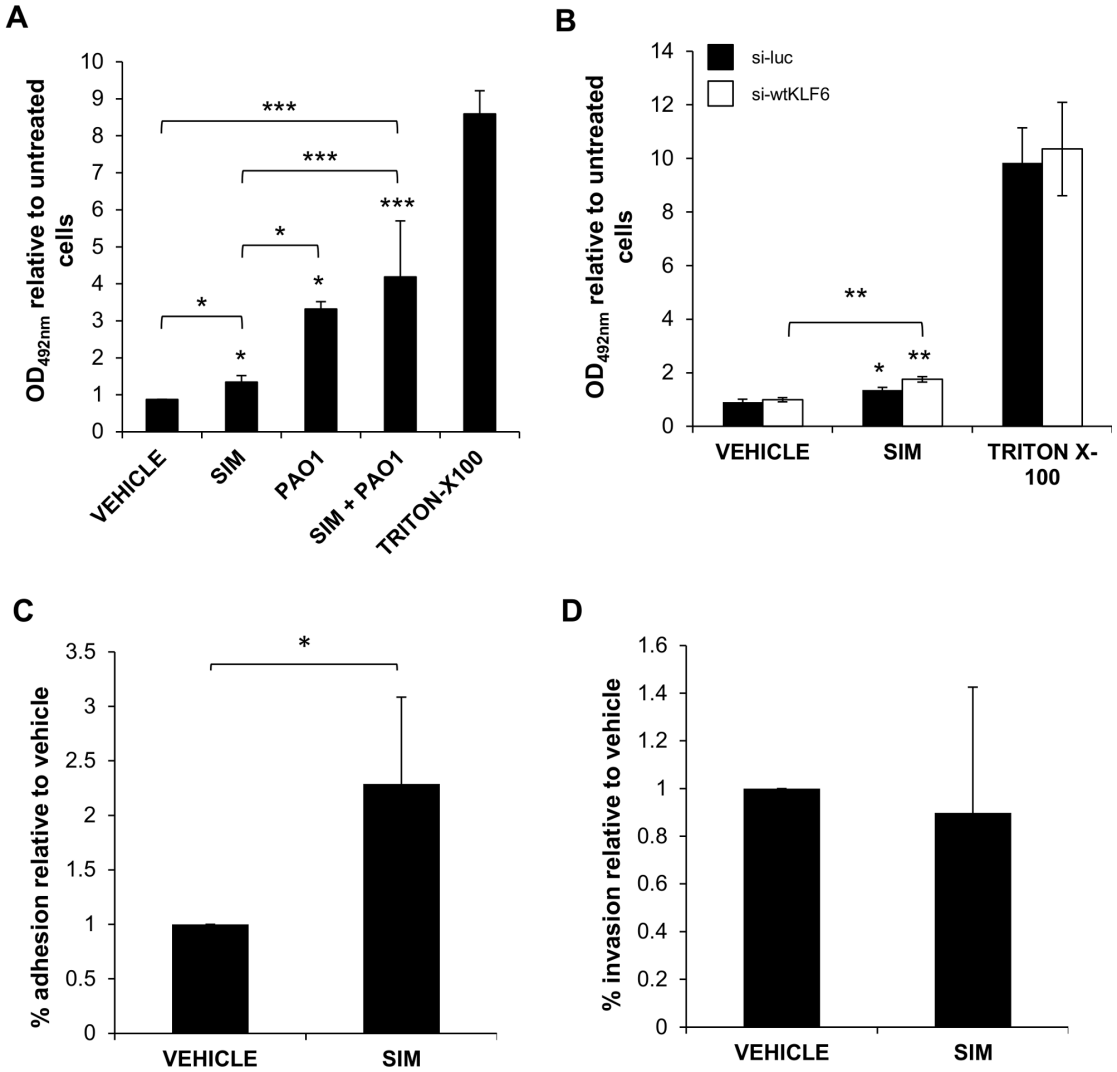
Cellular cytotoxicity and *P. aeruginosa* adhesion and invasion in response to simvastatin treatment. Cells were treated with 10 µM simvastatin for 24 hours (**A, B, C**), following which they were infected with *P. aeruginosa* PAO1at MOI 50∶1 (**A, C**). (**A**) Simvastatin significantly induced cytotoxicity but did not alter *P. aeruginosa*-mediated LDH release at 6 hours post infection. (**B**) Simvastatin-induced cytotoxicity was not significantly altered in si-wtKLF6 knockdown cells demonstrating wtKLF6 was not implicated in simvastatin-mediated cytotoxicity of lung epithelial cells. (**C**) Simvastatin treatment increased *P. aeruginosa* adhesion to cells, but (**D**) did not influence invasion at 3 hours of infection. *P, ≤0.05, **P, ≤0.01, *** *P*, ≤0.001.

Cytotoxicity was quantified by measuring lactate dehydrogenase (LDH) release from cells as a marker of tissue breakdown during statin treatment and *P. aeruginosa* infection (Figure 4A). Interestingly, at 6 hours post infection, we observed that simvastatin alone had a low but significant cytotoxic effect compared to untreated and DMSO treated cells respectively (*P* = 0.018, *P* = 0.04). While *P. aeruginosa* was significantly cytotoxic compared to untreated cells (*P* = 0.027) and statin-treated cells (*P* = 0.044), in simvastatin-treated cells *P. aeruginosa* infection was significantly more cytotoxic compared to untreated cells (*P* = 0.0003), vehicle-treated cells (*P* = 0.0006) and simvastatin-treated, uninfected cells (*P* = 0.0005). This sustained level of cytotoxicity between infected cells and statin-treated infected cells suggested that the presence of simvastatin does not attenuate cell damage caused by *P. aeruginosa*. LDH release was also examined at earlier intervals (1.5 and 3 hours post infection), but a significant level of cytotoxicity was not observed at these times (data not shown).

The role of wtKLF6 in inhibiting cell proliferation and inducing apoptosis has been extensively established [25], [26], [53]. We thus investigated whether wtKLF6 played a role in the cytotoxic effects exerted by simvastatin on lung epithelial cells. si-wtKLF6 and vector control A549 cells were treated with either 10 µM simvastatin or DMSO vehicle for 24 hours in serum-free medium, and cytotoxicity was quantified by measuring LDH release. In comparison to untreated cells, simvastatin caused a significant but comparable increase in LDH release in both vector control (*P* = 0.04), and si-wtKLF6 (*P* = 0.005) cells (Figure 4B). This demonstrated that wtKLF6 does not play a role in statin-mediated cytotoxicity and cell death, thus suggesting that wtKLF6-induced CCL20 and iNOS do not regulate these processes.

The effect of simvastatin on bacterial adhesion and invasion was also examined in A549 cells treated with 10 µM simvastatin for 24 hours and subsequently infected with *P. aeruginosa* PAO1 for 3 hours. Simvastatin increased the adhesion of PAO1 to A549 cells compared to those treated with vehicle (Figure 4C), but, in contrast, statin treatment did not alter invasion of *P. aeruginosa* compared to vehicle-treated cells (Figure 4D).

## Discussion

**Figure 5:**
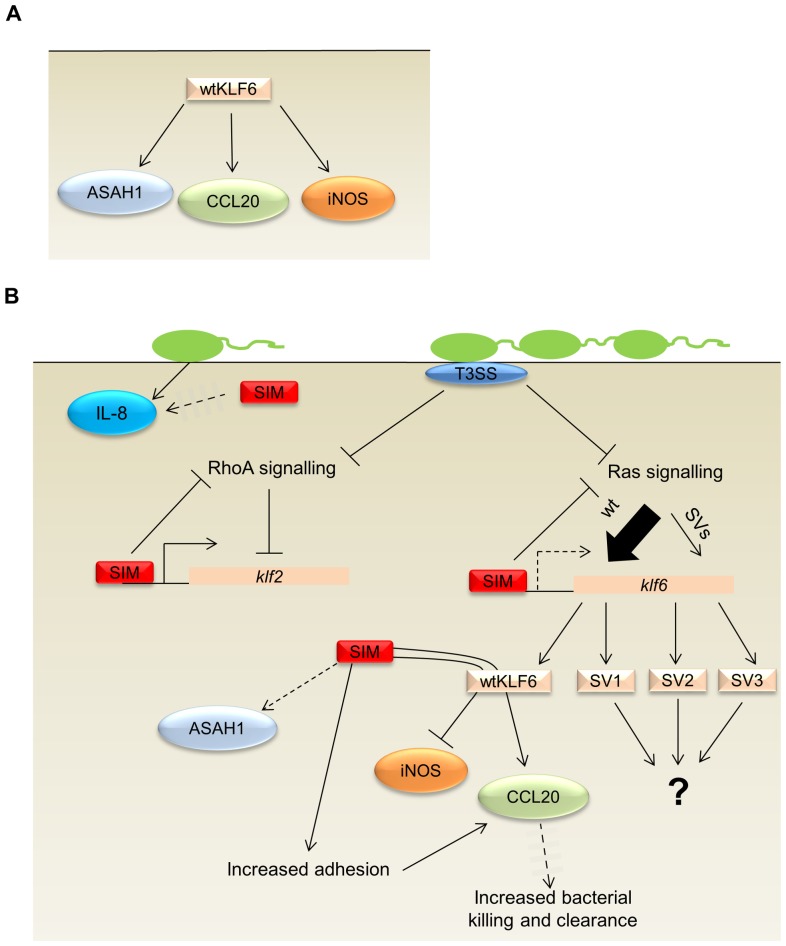
A model for the findings observed in this study. (**A**) In this work, wtKLF6 was found to regulate the expression of ASAH1, CCL20 and iNOS in lung cells in the absence of simvastatin (SIM).(**B**) SIM may induce KLF2 and KLF6 splice variant expression by binding to promoter elements and inhibition of Rho and Ras GTPase signalling. Rho and Ras GTPase signalling are also attenuated by the T3SS of *P. aeruginosa*, which may account for the induction of KLF2 and KLF6 by this species. The mechanism by which SIM induces IL-8 is unclear. However, the synergistic effect observed on CCL20 in this study may be a result of increased bacterial adhesion by SIM, and induction via wtKLF6. In addition, the wtKLF6-dependent reduction of iNOS by SIM may potentially be responsible for the observed increase in bacterial adhesion, Broken arrows represent mechanisms which require further elucidation.

In this study, we have demonstrated that simvastatin can modulate the expression of genes involved in the host response to *P. aeruginosa* infections in lung epithelial cells. A model for the mechanism behind these interactions is proposed in Figure 5. Previous studies have shown that attenuation of pro-inflammatory cytokine production is a key pleiotropic effect of statins in several tissue types (reviewed in [54] and [55]), including bronchial epithelial cells [56], while Kiener and colleagues showed that statins could increase monocytic pro-inflammatory cytokine and chemokine production in a mevalonate-dependent manner, thus sensitising them to challenge by inflammatory agents [15]. The latter of these studies correlates with our observation whereby simvastatin treatment induced IL-8 and CCL20 expression and that this was sustained and synergistically increased respectively upon infection with *P. aeruginosa*. The mechanism for this synergistic effect on CCL20 expression is unknown but we could hypothesise that it may be partly due to the increase in bacterial adhesion in the presence of simvastatin, as CCL20 expression in bacterial infections is linked to this phenotype [50]. However, *P. aeruginosa* adherence has also been linked to the induction of IL-8 [50], which was not synergistically induced in this study. Therefore, other factors are likely to be involved in the additive induction of CCL20 by *P. aeruginosa* and simvastatin. Furthermore, *P. aeruginosa*-mediated IL-8 and CCL20 production has been shown to be TLR5-dependent [43], [52], but TLR5 does not appear to be involved in the simvastatin induction of these factors, as TLR5 expression was not altered by statin treatment. In this study, we also showed the novel alteration of ASAH1 and iNOS expression in response to simvastatin treatment. The induction of ASAH1 by simvastatin could potentially be beneficial, as it may decrease ceramide-mediated inflammation in people with CF and other inflammatory disorders. On the other hand, decreased iNOS expression in CF patients is associated with increased bacterial adherence [39], and the simvastatin-mediated reduction of iNOS expression that was observed in si-luc-transfected cells (Figure 3E) and untransfected A549 cells (data not shown) may potentially be responsible for the increased adhesion of *P. aeruginosa* in the presence of this compound.

Our results correlate with previous reports of statin induction of KLF2 [22], [23] and we demonstrate that simvastatin had a significantly greater affinity for KLF2 induction than *P. aeruginosa*. This is possibly because statins have been shown to directly induce KLF2 expression by binding to a MEF2 transcription factor site in the *klf2* promoter region [22], while the induction of KLF2 by *P. aeruginosa* is proposed to be indirectly regulated by Type 3 toxin-mediated inhibition of host protein (RhoA) activity [17], [57], [58]. In contrast to KLF2, KLF6 has not previously been shown to be induced by statins. Here we demonstrate for the first time that simvastatin did induce KLF6 in airway epithelial cells. Furthermore, as *in silico* prediction of binding sites in the KLF6 promoter region demonstrates that a putative MEF2A binding site is located 228 bp upstream of the *klf6* start site (data not shown), we propose that statins may induce *klf6* transcription in a similar manner to *klf2*. However, this does not account for the induction of KLF6 alternative splicing by statins. Production of KLF6 splice variants is regulated by a signalling cascade which involves the Rho GTPase Ras and the downstream Akt and PI3-K signalling proteins [28]. Previous studies have demonstrated that statins can modulate the phosphorylation and activation of the Akt signalling molecule in the Ras pathway [59], [60], which may account for splice variant production by simvastatin. However, this may be balanced with the characteristic statin-mediated inhibition of Ras prenylation first described by Leonard *et al*. [61].

We also identified for the first time the induction of KLF6 alternative splicing by *P. aeruginosa*. Previously we reported that KLF6 is induced by *P. aeruginosa* T3SS toxins [17], and we propose that the induction of KLF6 splice variants by *P. aeruginosa* may be mediated through 2 T3SS-dependent mechanisms. Firstly, *P. aeruginosa* may induce KLF6 alternative splicing by modulating Ras signalling as the ExoS toxin of *P. aeruginosa* has been found to regulate activation of Ras signalling [62]. Furthermore, the *P. aeruginosa* T3SS also induces the production of reactive oxygen species (ROS) during infection [63] and these compounds can induce KLF6 alternative splicing [64]. It was interesting to note that wtKLF6 was the dominant KLF6 variant in A549 lung epithelial cells both at basal level and in response to *P. aeruginosa* infection, in spite of them being a cancer cell line. Previously, increased SV1 has been linked to various cancers including that of the lung. Nevertheless our results clearly demonstrate a role for wtKLF6 in modulating the host response to infection.

wtKLF6 was also found to regulate the statin effects on CCL20 and iNOS but not ASAH1 in lung epithelial cells. Interestingly, our data showed that wtKLF6 did not appear to regulate the expression of PPARγ in lung cells as it did in kidney cells [40], suggesting that this interaction and subsequent CCL20 induction may be tissue-specific. The regulation of statin-mediated CCL20 and iNOS induction by wtKLF6 affirms the role of this protein in the immune response, and suggests that wtKLF6 has a role as a regulator of immune signalling. However, wtKLF6-dependent ASAH1 expression suggests that, like statins, wtKLF6 may induce both pro- and anti-inflammatory responses, and may thus play a role in maintaining a balanced inflammatory response. Furthermore, KLF6 splice variants may also antagonise the pro-inflammatory effects of wtKLF6; SV1 has been found to have a negative regulatory effect on the pro-inflammatory cytokine TNFα, whereas wtKLF6 may induce this protein [64].

Statins have been proposed as novel therapeutics in the fight against infections [1], [2], and this work demonstrates some potential effects simvastatin could have if used as an anti-microbial agent in respiratory infections. The manipulation of pro-inflammatory cytokines and KLFs by simvastatin may lead to improved survival and bacterial clearance mediated by IL-8 and CCL20, whereas statin-induced KLF2 may serve as a regulator to downplay other elements of the inflammatory response. The dominance of wtKLF6 compared to the other splice variants suggests that a general anti-proliferative effect is conferred on simvastatin-treated and *P. aeruginosa* infected cells. Further studies are needed to elucidate the mechanistic role of KLF6 splice variants in the *P. aeruginosa*-host response, and to determine whether statins influence the expression of downstream splice variant targets.
